# Five years of *Scientific Data*

**DOI:** 10.1038/s41597-019-0065-y

**Published:** 2019-05-28

## Abstract

*Scientific Data* published its first batch of papers five years ago this week. Here, we reflect on our progress and thank all those that have helped us along the way.

Over the last five years, funders and journals around the globe, and across a wide range of disciplines, have adopted new policies that better recognize and promote data sharing. Researchers themselves have rallied behind the FAIR Data Principles^1^, hosting workshops and training events that aim to make research data more findable, accessible, interoperable and reusable.

There have, of course, also been differences of opinion and obstacles. Researchers who rely heavily on the data of others were accused of being ‘research parasites’^2^, a label that some chose to embrace and transform into a ground-breaking award that celebrates innovative data reuse^3^. Other researchers have had to contend with shifting public views caused by high-profile data scandals and an increasingly divisive political climate.

Against this backdrop, *Scientific Data* has sought to be an advocate not just for open data sharing, but also for responsible and effective data sharing. Five years since our launch, over 750 data descriptors have been published at the journal, releasing and describing datasets across a wide range of fields and topics. We are delighted to see that our papers have been collectively cited by more than 6000 other scholarly papers, many of which are themselves compelling examples of data reuse.

We would like to thank all of our authors who over these last five years have supported *Scientific Data*. We also extend our sincerest thanks to our many dedicated and hardworking Editorial Board members and peer reviewers, along with the members of our new Senior Editorial Board (https://go.nature.com/2XFatD6). The journal never would have made it this far without all of your hard work and support.

Equally important has been the support of the many data repositories with whom we work. There are now over 100 repositories on our recommended list (http://go.nature.com/2eLHBFP), and we work with an even wider range of institutional and project-specific repositories. Because of the diversity of these systems and their policies, our staff often correspond extensively with submitting authors and the hosting repository to find the right way to host our authors’ data and to share it securely with our referees. We would like to extend our thanks to the many repository managers and curators who have borne with us when things have become complicated, which does happen, especially for the complex datasets that are common at the journal.

Many types of data submitted to the journal do not have a dedicated specialist data repository. For authors of such datasets, we have built strong partnerships with a number of ‘generalist’ data repositories, most notably Figshare (http://figshare.com) and Dryad (https://datadryad.org/). About a third of the journal’s publications use one of these repositories to help host at least part of their data.

We would also like to extend a very special thinks to the researchers behind ISA-tools (http://isa-tools.org/) and FAIRsharing^4^. Both projects are led by the group of Susanna-Assunta Sansone, *Scientific Data’s* Honorary Academic Editor. FAIRsharing (https://fairsharing.org/) is a curated portal that tracks and interlinks community reporting standards, databases, repositories and data policies. As a result of a long-standing collaboration, users can browse our recommended repositories through a dedicated collection (https://fairsharing.org/recommendation/ScientificData), and use FAIRsharing’s tools to browse key information and discover related reporting standards. *Scientific Data* uses the ISA framework as an integral part of our unique metadata curation process^5^.

Beyond the journals’ own pages, *Scientific Data* has led or been a part of a number of initiatives across the wider publishing and research community that promote best practice on topics like sharing clinical research data^6,7^, data citation^8^ and research data policies^9^. The journal has also forged partnerships with other publications to share best practice and enable the publication of more reproducible research, most notably journals in the Nature Research family^10^.

The journal also continues to engage with the research community in creative ways, and our conference Better Science through Better Data, will be holding its sixth event in the fall of 2019.

To celebrate our achievements on our 5^th^ anniversary, we have launched a special web page with an interactive history of the journal’s most important milestones. We invite you to explore and share it (https://www.nature.com/sdata/5th-anniversary).

Here’s to the next five exciting years of data.
